# Low-frequency deep brain stimulation reveals resonant beta-band evoked oscillations in the pallidum of Parkinson’s Disease patients

**DOI:** 10.3389/fnhum.2023.1178527

**Published:** 2023-09-22

**Authors:** Valentina Zapata Amaya, Joshua E. Aman, Luke A. Johnson, Jing Wang, Remi Patriat, Meghan E. Hill, Colum D. MacKinnon, Scott E. Cooper, David Darrow, Robert McGovern, Noam Harel, Gregory F. Molnar, Michael C. Park, Jerrold L. Vitek, David Escobar Sanabria

**Affiliations:** ^1^Department of Neurology, University of Minnesota, Minneapolis, MN, United States; ^2^Department of Radiology, University of Minnesota, Minneapolis, MN, United States; ^3^Department of Neurosurgery, University of Minnesota, Minneapolis, MN, United States

**Keywords:** stimulation-evoked beta responses, Parkinson’s disease, deep brain stimulation, beta-oscillations, globus pallidus, circuit resonance

## Abstract

**Introduction:**

Evidence suggests that *spontaneous* beta band (11–35 Hz) oscillations in the basal ganglia thalamocortical (BGTC) circuit are linked to Parkinson’s disease (PD) pathophysiology. Previous studies on neural responses in the motor cortex evoked by electrical stimulation in the subthalamic nucleus have suggested that circuit resonance may underlie the generation of spontaneous and stimulation-evoked beta oscillations in PD. Whether these stimulation-evoked, resonant oscillations are present across PD patients in the internal segment of the globus pallidus (GPi), a primary output nucleus in the BGTC circuit, is yet to be determined.

**Methods:**

We characterized spontaneous and stimulation-evoked local field potentials (LFPs) in the GPi of four PD patients (five hemispheres) using deep brain stimulation (DBS) leads externalized after DBS implantation surgery.

**Results:**

Our analyses show that low-frequency (2–4 Hz) stimulation in the GPi evoked long-latency (>50 ms) beta-band neural responses in the GPi in 4/5 hemispheres. We demonstrated that neural sources generating both stimulation-evoked and spontaneous beta oscillations were correlated in their frequency content and spatial localization.

**Discussion:**

Our results support the hypothesis that the same neuronal population and resonance phenomenon in the BGTC circuit generates both spontaneous and evoked pallidal beta oscillations. These data also support the development of closed-loop control systems that modulate the GPi spontaneous oscillations across PD patients using beta band stimulation-evoked responses.

## 1. Introduction

Parkinson’s disease (PD) affects multiple circuits in the basal ganglia thalamocortical (BGTC) network involved in motor control. Evidence suggests that high-power, spontaneous local field potential (LFP) oscillations in the beta band (11–35 Hz) in the BGTC circuit are associated with the progression of parkinsonism and the manifestation of rigidity and bradykinesia in PD patients (Brown et al., 2001; Brown, 2003; Brown and Williams, 2005; Kühn et al., 2006, 2009; Chen et al., 2007; Little et al., 2012). These spontaneous beta oscillations are particularly prominent in the subthalamic nucleus (STN) and internal segment of the globus pallidus (GPi) (Brown et al., 2001), which are the primary targets for deep brain stimulation (DBS) therapy in PD. Low-frequency electrical stimulation pulses in the STN, delivered via DBS leads, have been shown to evoke beta-band responses over the primary motor cortex (Baker et al., 2002; MacKinnon et al., 2005; Devergnas and Wichmann, 2011; Walker et al., 2012; Zwartjes et al., 2013; Miocinovic et al., 2018) and in the STN (Campbell et al., 2022). The resonance of the beta band evoked responses in the motor cortex (MC) has been shown to decrease when antiparkinsonian medication (levodopa) is delivered to PD patients (Eusebio et al., 2009). These data support the concept that resonance—the susceptibility of a circuit to oscillate at its natural frequency—is involved in the generation of beta oscillations in the sub-thalamocortical circuit in PD. Understanding whether stimulation in the basal ganglia evokes beta-band resonant oscillations in the basal ganglia is needed to characterize how beta oscillations are generated and propagated in the BGTC circuit in PD.

Beyond using stimulation-evoked beta band responses to characterize circuit dysfunction in PD, they have also been proposed for the development of closed-loop, real-time neural control systems (Escobar Sanabria et al., 2020, 2022; Sanabria et al., 2022). In a proof-of-concept study with a PD patient, Escobar Sanabria et al. (2022) showed that stimulation-evoked beta oscillations in the GPi can be used to suppress or amplify spontaneous beta-band oscillations in the GPi when electrical pulses are delivered with precise amplitude and timing. This technique, referred to as evoked interference closed-loop DBS (eiDBS), is suitable to modulate frequency-specific neural activity and characterize the causal role of controlled changes in beta-band activity in the manifestation of PD. The feasibility of implementing eiDBS in the GPi across PD patients is yet to be determined. Understanding whether resonant, stimulation-evoked beta-band oscillations are present across PD patients in the GPi, is the first step toward determining this feasibility.

Here, we demonstrate that low-frequency (<3 Hz) stimulation in the GPi evokes long-latency (>50 ms) beta-band neural responses in the GPi in four PD patients (five hemispheres). We also show that the frequency and spatial localization of the neural sources generating both stimulation-evoked and spontaneous beta oscillations are correlated. These data support the hypothesis that the same neuronal pool and resonance phenomenon generates both spontaneous and evoked pallidal beta oscillations. Our data also provide evidence that beta-band stimulation-evoked responses can be employed to modulate GPi spontaneous oscillations across PD patients using closed-loop control systems (e.g., eiDBS).

## 2. Materials and methods

### 2.1. DBS lead externalization procedure

**Figure 1:**
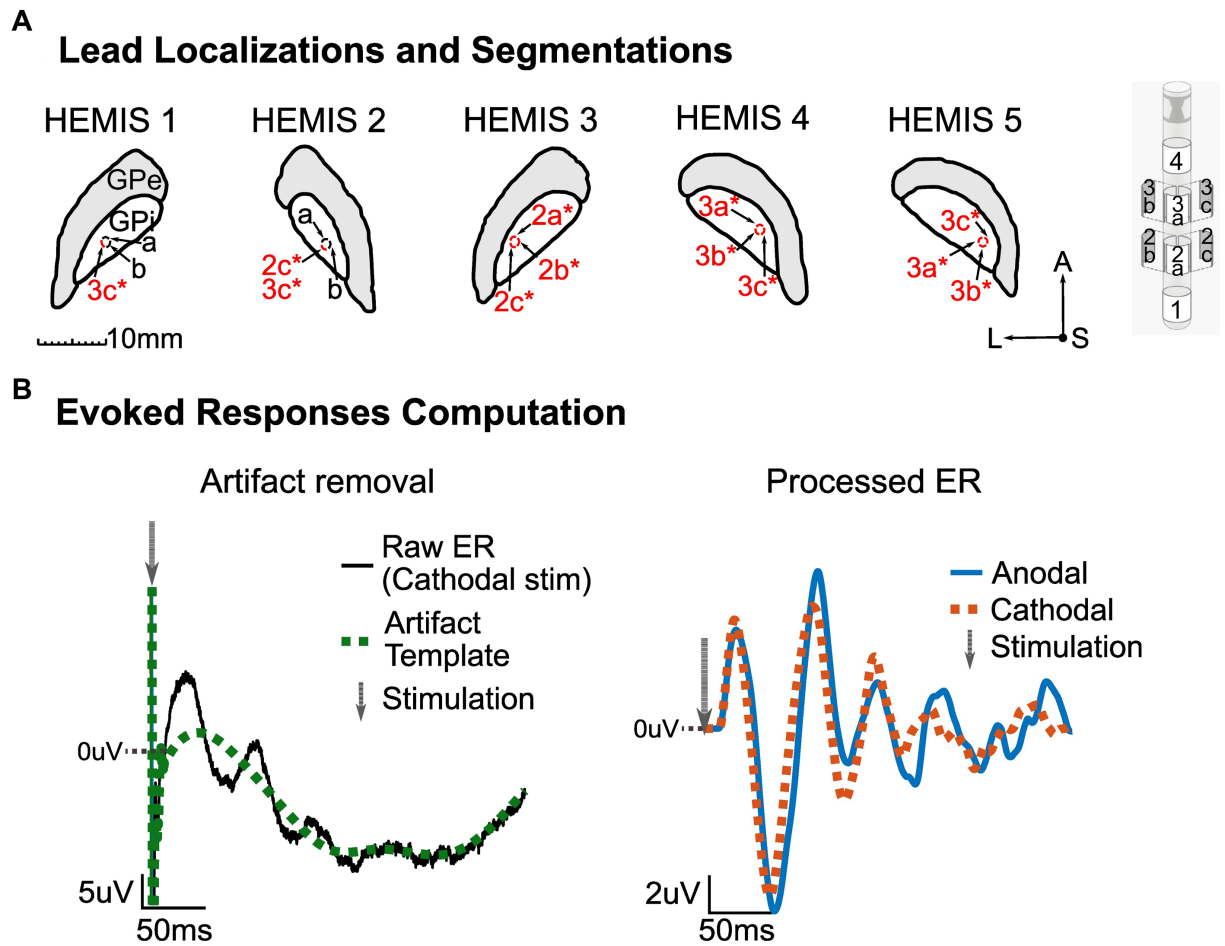
**(A)** Left: MRI segmentation of the GPi with corresponding lead localization and stimulation channels (marked in red and with an asterisk) for each hemisphere. Center: Segmentations’ orientation-A, Anterior; S, Superior/Dorsal; L, Left. Right: Abbott Infinity model 6172 lead. All images shown here are on a lateral-anterior plane (horizontal sections). **(B)** Illustration of ER computation methodology. Left: Artifact removal procedure with raw averaged potential (black) and artifact template (green, dotted). The gray arrow shows the stimulation time. Right: processed ER in anodal (blue) and cathodal (red, dotted) stimulation configurations. The gray arrow shows the stimulation time. The zero line indicates zero-volts, and the values above this line are positive. The DC offset of the pre-stimulus data segment in the stimulation-evoked responses was removed.

All patient procedures were approved by the University of Minnesota Institutional Review Board (IRB#1701M04144), with consent obtained according to the Declaration of Helsinki. Four patients with idiopathic PD implanted with DBS leads in the GPi participated in this study. We recorded from the two hemispheres in one patient to obtain five hemispheres in total. Hemispheres HEMIS 2 and HEMIS 3 correspond to these two hemispheres. DBS lead targeting and postoperative lead localization were performed using magnetic resonance imaging (MRI) with 3T or 7T scanners (Duchin et al., 2018; Patriat et al., 2018). Electrophysiological mapping techniques (Vitek et al., 1998) were employed to identify the sensorimotor region of GPi during implantation surgery. All patients were implanted with a directional “1–3–3-1” electrode (hemispheres 1, 2, and 3 with Abbott Infinity model 6172, and hemispheres 4 and 5 with Boston Scientific Vercise Cartesia model DB-2202-45). Figure 1A illustrates the location of the DBS leads relative to the GPi. After implantation, the lead extension was tunneled to a subcutaneous pocket in the chest and then connected to a research-dedicated extension that was externalized through an abdominal incision (Aman et al., 2020). Recordings from the DBS lead occurred 4–8 days after DBS implantation surgery. After data were collected, the patients returned to the hospital, where the percutaneous (research) extension was removed, and the implantable pulse generator (IPG) was placed in the chest pocket.

### 2.2. Neurophysiological data acquisition

Deep brain stimulation leads were connected to an ATLAS neurophysiological recording system (Neuralynx, Bozeman, MT, United States) via customized connectors to record LFPs from the GPi in the presence and absence of low-frequency electrical stimulation. The ATLAS amplifiers have a large input range (±132 mV) and a high sampling rate (24K Samples/s) that enabled us to recover the shape of short-duration stimulation artifacts and record neural data in the same brain region where stimulation was delivered. We used EEG scalp electrodes placed along the midline as a reference and ground for the DBS lead recordings. Spontaneous LFPs were collected during a period of 5.38 ± 0.09 min while the patient was sitting looking at a fixed point (black plus sign) in front of them.

We used two neurostimulation systems to deliver low-frequency stimulation for the collection of stimulation-evoked response (ER) data. In two hemispheres (HEMIS 1 and HEMIS 2), we employed the NeuroOmega mapping system (Alpha Omega, Nazareth, Israel) to deliver current-controlled electrical stimulation. In hemispheres HEMIS 3, HEMIS 4, and HEMIS 5, we used the g.Estim neurostimulator (g.tec, Schiedlberg, Austria). Biphasic, symmetric, charge-balanced waveforms were delivered in both cathodal and anodal (reversed polarity) configurations. A patch, skin-surface electrode, was placed on the patient’s chest for the current return in the monopolar montage. The monopolar or bipolar stimulation montages and corresponding amplitudes used for analysis in each hemisphere are reported in Table 1. The stimulation montages that evoked responses with the highest signal-to-noise ratio were selected for analysis. We delivered stimulation pulses with a frequency less than or equal to 3 Hz. Table 1 indicates the times when the stimulation-related recordings took place relative to the times when the patients took their levodopa medication. These times are reported here since levodopa may influence the amplitude of the ERs, as discussed in the Limitations section.

**Table 1:** DBS lead manufacturer, target hemisphere, stimulation parameters, and time when the data were acquired relative to medication administration for each hemisphere.

Hemisphere ID	Lead	Target	Time after meds	Stim channels	Stim amplitude	Recording montage	Number of differential channels used in spatial correlation	Stimulator
HEMIS 1	Abbott	Left GPi	12 min	3c-patch	3 mA	2a–3a	N/A (no clear beta peak in ASD)	Neuro- Omega
HEMIS 2 (same patient as HEMIS 3)	Abbott	Right GPi	150 min	2c-3c	3 mA	2b–4	N/A (only one channel with a clear stimulation-evoked response due to long-lasting stimulation artifacts)	Neuro- Omega
HEMIS 3 (same patient as HEMIS 2)	Abbott	Left GPi	>8 h	2abc-patch	2 mA	1–4	6	g.Estim
HEMIS 4	Boston Scientific	Right GPi	11 min	3abc-patch	3 mA	2a–4	7	g.Estim
HEMIS 5	Boston Scientific	Right GPi	>8 h	3abc-patch	3 mA	2a–4	6	g.Estim

*Boston Scientific contacts 5, 6, and 7 correspond to 3a, 3c, and 3b, respectively.

### 2.3. Data analysis and statistics

#### 2.3.1. Spectral characteristics of spontaneous LFPs

All analyses were performed using customized scripts in MATLAB (MathWorks, 2019). Differential recordings were computed from spontaneous LFP of non-stimulating channels across ring levels. These differential potentials were band-pass filtered using a bandpass Butterworth filter with cut-off frequencies 0.1 and 500 Hz. Amplitude spectral density (ASD) curves were calculated from 5 s segments of the filtered spontaneous LFP using the Welch method (*pwelch* command in MATLAB). The ASD at each frequency is equal to the square root of the PSD. The median ASD across ASD curves derived from the 5 s LFP segments was used for the analyses. To compute the ASD curves, we used 2^16^ discrete Fourier transform points, a Hamming window with 2^13^ points, and an overlap of 2^12^ samples (50% overlap). The 1/f trend of these curves was removed using a template approach in the frequency domain to compare the relative differences in the amplitude of LFPs in the beta band across recording channels (Corbit et al., 2007; Ouyang et al., 2020; Gerster et al., 2022). Briefly, we constructed an interpolated signal that followed the trend of the ASDs at low (0.25–5 Hz) and high frequencies (40–200 Hz) and removed this interpolated trend from the original ASD curve to obtain the de-trended ASD curves for analysis. Of note, the ASD was used instead of the power spectral density (PSD) to determine linear correlations (instead of quadratic) between the amplitudes of spontaneous and stimulation-evoked potentials across recording sites. We sought to determine linear relationships because they are more straightforward to visualize and characterize than nonlinear ones.

#### 2.3.2. Stimulation-evoked responses

ERs for both cathodal and anodal pulses were computed by averaging differential LFP segments aligned with stimulation (−100 to +300 ms around the stimulation peak). We collected 523 ± 291 LFP segments in each of the studied hemispheres for the ER computation. To suppress the stimulation artifacts, we used a template subtraction method (Campbell et al., 2022; Escobar Sanabria et al., 2022). Briefly, we used the averaged data segments to create templates of the electric artifact (anodal and cathodal) and obtained the stimulation responses (neural response + artifact). The artifacts were characterized by a short-latency, high-frequency component followed by a low-frequency drift (Figure 1B). To compute the artifact templates, we did the following:

1. Inspected the anodal and cathodal LFP averages and confirmed the presence of neural responses whose polarity was invariant to the stimulation setting (anodal vs. cathodal). This nonlinear phenomenon is specific to neural responses but not to electrical artifacts (Baker et al., 2002).
2. Employed the mean between the averaged cathodal and anodal ERs to minimize the amplitude of the artifacts.
3. Selected a sample time (n_0) when the short-latency artifacts ended.
4. Visually selected samples along the LFP averages to interpolate the template model so that low-frequency drifts were captured.
5. Interpolated the data from sample (n_0) to the final sample in the averaged data segment using a piecewise cubic interpolation (spline command in MATLAB, MathWorks, 2019).
6. Set the template’s first (n_0) samples equal to the averaged segment to preserve the short-latency artifacts in the artifact template model.

The ERs were computed by subtracting the artifact template from the average data segments. Figure 1B shows an example of the artifact template and neural evoked responses (cathodal and anodal).

#### 2.3.3. Spectral characteristics of stimulation-evoked responses

The amplitude of ERs in the time and frequency domain was characterized using the continuous wavelet transform (*cwt* command in MATLAB with the analytic Morse wavelet).

#### 2.3.4. Statistical assessment of stimulation-evoked responses

Surrogate ERs were computed from spontaneous LFPs as a control condition. The surrogates were calculated by averaging differential LFP segments aligned with randomly generated time samples (−100 to +300 ms around the selected sample). The resulting averages underwent the same artifact removal and signal processing described above for the ERs. A permutation test without replacement was performed to assess whether the amplitude of the detected ERs was the result of chance (Romano and Tirlea, 2022). We evaluated whether the amplitude at each frequency and time point was significantly greater than the chance distribution via a permutation test based on the LFP surrogate ERs. *p*-values were corrected with the False Discovery Rate (FDR) method for multiple comparisons across frequencies and times (Benjamini and Hochberg, 1995). Clusters of wavelet amplitudes with corrected *p* < 0.05 were considered significant.

#### 2.3.5. Spatial correlation between the locations where spontaneous and stimulation-evoked beta oscillations were generated

To determine whether the location of neural sources generating spontaneous beta-band activity matched the location where beta-band ERs originated, we studied the correlation between scalar measures of the ASDs (spontaneous activity) and the wavelet transform (ERs) across recording montages (sensing sites). The ASD scalar measures were equal to the sum of ASD values in the band centered at the peak ASD frequency (4 Hz bandwidth). The wavelet scalar measures were equal to the sum of wavelet spectrogram values in the band centered at the peak frequency (4 Hz bandwidth) and time. Correlations between the ASD and wavelet scalar measures across differential electrode recordings were evaluated using the Pearson correlation coefficient. A high correlation indicates that monopolar or dipolar current sources producing the electric potentials associated with the spontaneous activity, and ERs are located within the same region and are likely linked to the same neuronal population. The rationale behind this argument is that two current sources (dipolar or monopolar) located within the same region in a volume conductor generate the same electric potential profile in space (Poisson equation of electrostatics) (Nunez and Srinivasan, 2006; Griffiths, 2017). This correlation analysis excluded differential LFP channels with elevated noise and small signal-to-noise ratios that did not allow us to characterize spontaneous activity or ERs. This exclusion was based on our visual inspection of the raw, spontaneous LFP data, ASD curves, ER time series, and ER wavelet spectrograms. We excluded spontaneous LFP channels with elevated wide-band noise observed in the time series and ASD curves. We also excluded LFP channels in which noise and stimulation-induced electrical artifacts limited our ability to observe and characterize stimulation-evoked neural activity even after applying the artifact suppression approach described above. In HEMIS 1 and 2, we excluded differential LFP channels due to the stimulation-induced artifacts’ long duration and elevated amplitude. In HEMIS 3, we excluded channels due to excessive noise levels in the spontaneous LFP recordings. The number of channels used for the spatial correlation analysis is reported in Table 1.

#### 2.3.6. Correlations between the frequencies of spontaneous and stimulation-evoked beta oscillations across subjects

To understand whether the resonant frequencies of the spontaneous oscillations and ERs matched across the studied PD hemispheres, we calculated the Pearson correlation between the peak frequencies of the ASDs (spontaneous activity) and wavelet spectrograms (ERs).

## 3. Results

### 3.1. Spontaneous LFP activity exhibited amplitude peaks in the beta-band in 4/5 hemispheres

**Figure 2:**
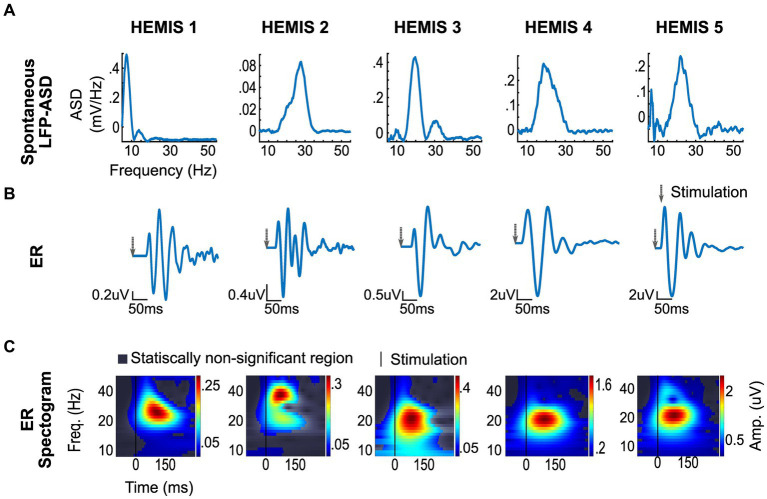
ERs and spontaneous (differential) LFP recording from selected montages in each hemisphere. **(A)** Top row: Spontaneous LFP-ASD curves after 1/f detrending. **(B)** Middle row: ER time series. The gray arrow indicates the stimulation time. **(C)** Bottom row: ER spectrograms (time-frequency maps). The vertical line shows stimulation time.

The ASD curves of the spontaneous LFPs exhibited a peak at 24 ± 4 Hz in 4/5 hemispheres. In one hemisphere (HEMIS 1), a beta peak in the spontaneous LFPs was not clearly identified. Figure 2 shows the ASD curves associated with the selected recording montage (from differential LFPs). Supplementary Figure S1 shows the ASD curves for each hemisphere and all montages that did not exhibit elevated noise to enable clear data visualization.

### 3.2. Stimulation pulses evoked neural responses in the beta-band in all hemispheres

The amplitude of the ERs was significantly greater than the chance distribution in the beta-band in all hemispheres. The mean latency from the stimulus to the peak of the ER was 42.5 ± 11.8 ms with a peak frequency of 23 ± 6 Hz (Figure 2).

### 3.3. Stimulation-evoked responses in the GPi oscillated at the peak frequency of the GPi spontaneous oscillations

**Figure 3:**
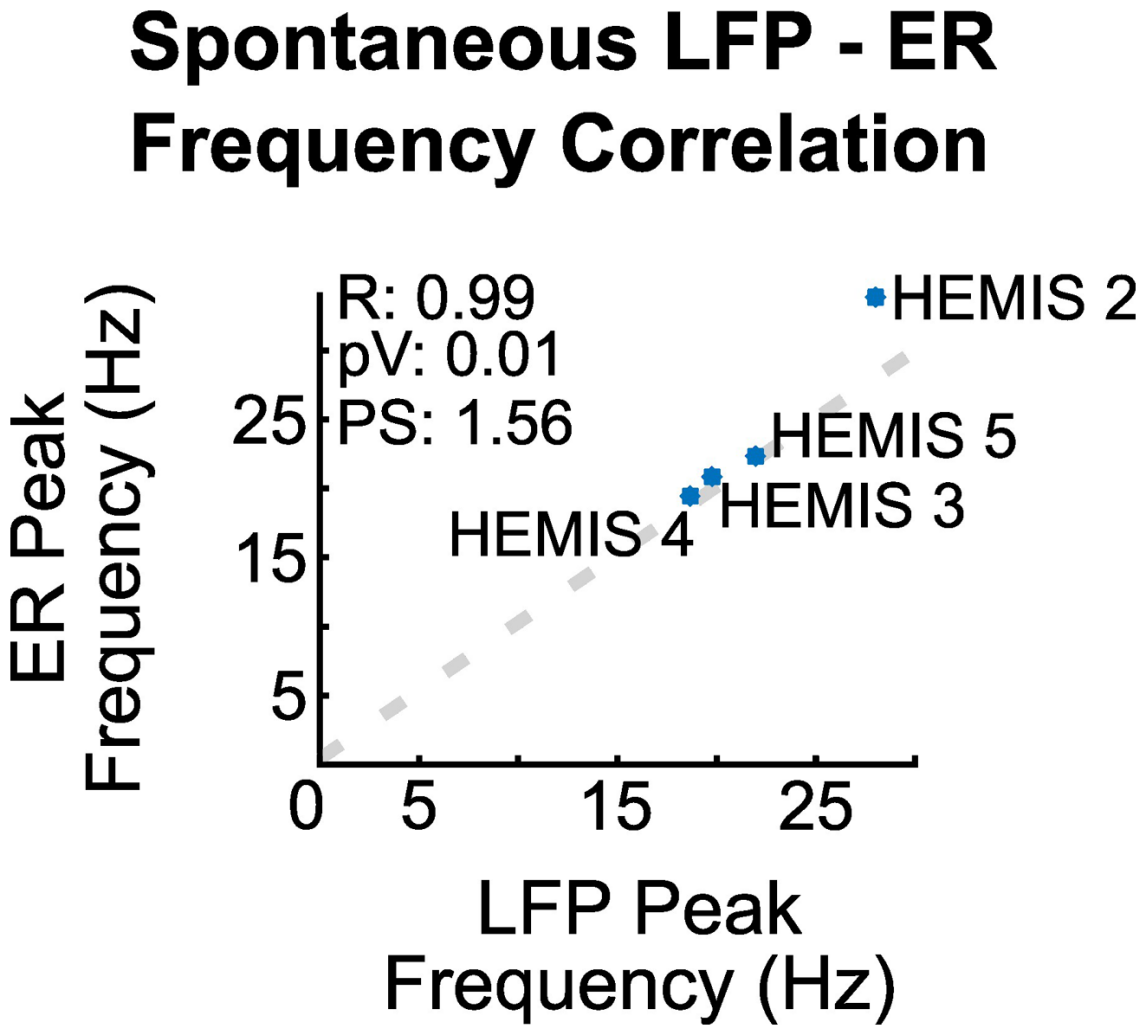
Correlation between the peak frequency of the ER spectrograms and the ASD curves of the spontaneous LFPs across hemispheres. R is the regression coefficient, pV is the value of p, and PS is the linear regression slope.

The peak -frequencies of the ER spectrograms and the LFP-ASDs were significantly correlated across the 4/5 hemispheres whose ASDs exhibited elevated beta-band activity (value of p: 0.01, R: 0.99). Figure 3 illustrates the scatter plot of the peak frequencies across these hemispheres.

### 3.4. The neural sources generating the spontaneous and stimulation-evoked beta oscillations were correlated in space

**Figure 4:**
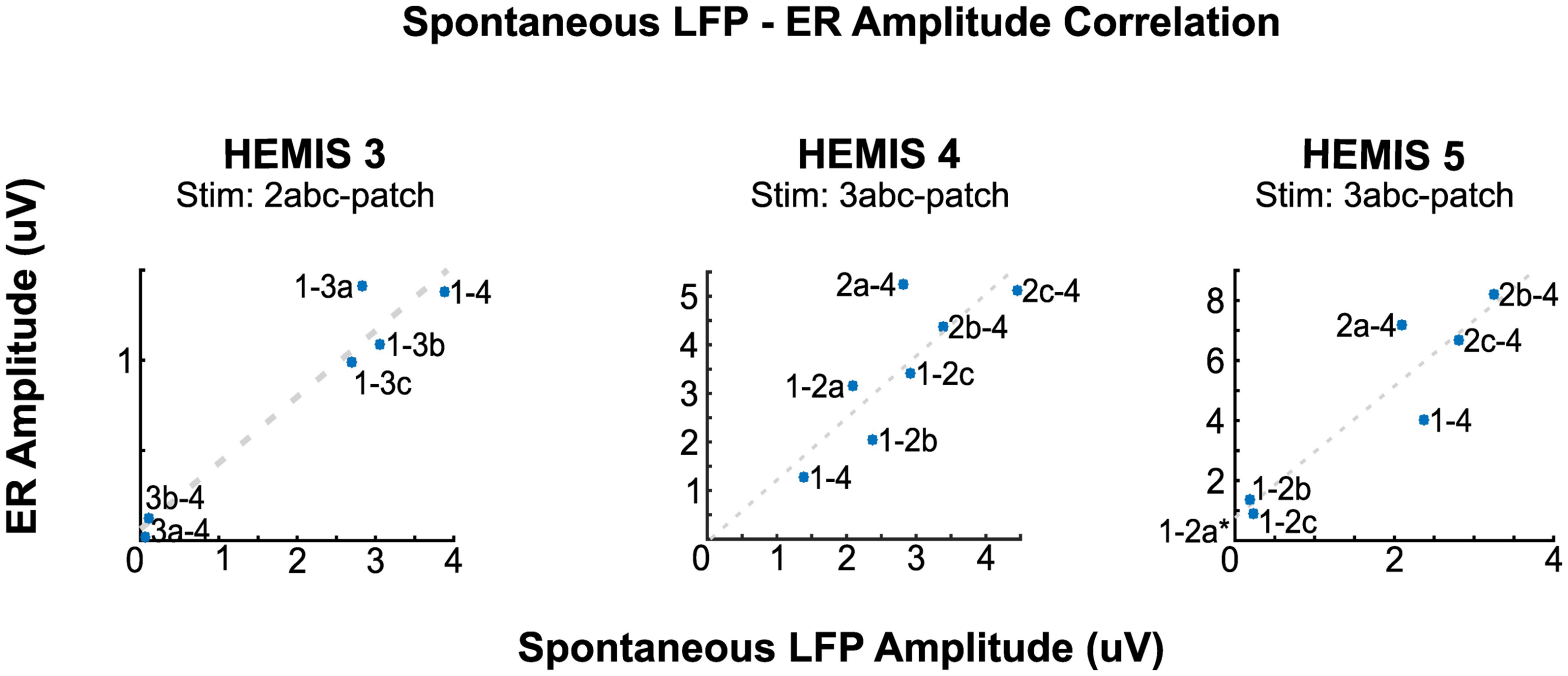
Correlation between the amplitudes of the ERs and spontaneous LFPs at the peak beta-band frequency for hemispheres HEMIS 3 (19–23 Hz), HEMIS 4 (17–21 Hz), and HEMIS 5 (19–23 Hz). Each point represents a differential potential. Regression coefficients (R), *p*-values (pV), and linear fit slope (PS) for each hemisphere were as follows. HEMIS 3: *R* = 0.97, pV = 0.001, PS = 0.37. HEMIS 4: *R* = 0.81, pV = 0.02, PS = 1.25. HEMIS 5: *R* = 0.94, pV = 0.001, PS = 2.19. The gray 1-2a label in HEMIS 5 represents a negative value that is out of the chart (Spontaneous LFP Amplitude: −0.0476, ER Amplitude: 0.7232). This amplitude value from the ASD curve is negative because of the 1/f curve subtraction described in Materials and methods section.

In hemispheres, HEMIS 3, HEMIS 4, and HEMIS 5, the maximum amplitudes of the ASD curves and ER spectrograms (beta-band) were correlated across differential potentials computed between pairs of DBS lead contacts not used for stimulation. The within-subject correlations were equal to 0.97, 0.80, and 0.92 for hemispheres HEMIS 3, HEMIS 4, and HEMIS 5, respectively (*p*-values were 0.0013, 0.023, and 0.0025). Figure 4 shows the scatter plots of ASD and ER spectrogram amplitudes in the beta-band in these hemispheres. Hemisphere HEMIS 1 did not exhibit a clear beta-band peak in the ASD curve; therefore, this hemisphere was excluded from the spatial correlation analysis. Due to the limited number of differential potentials with a large signal-to-noise ratio in hemisphere HEMIS 2, we excluded this hemisphere from the spatial correlation analysis. Supplementary Figure S1 shows the ERs and spontaneous LFP-ASD curves for each hemisphere and all montages that did not exhibit elevated noise.

## 4. Discussion

### 4.1. Existence of beta-band evoked responses in the GPi

Previous studies have shown that single bipolar stimulation pulses, delivered in the STN or GPi of PD patients at low frequency, evoked short latency and high-frequency as well as long latency and low-frequency evoked responses on scalp EEG recordings over the MC (Eusebio et al., 2008; Walker et al., 2012; Miocinovic et al., 2018; Romeo et al., 2019). More recent studies characterizing neural responses in basal ganglia (GPi and STN) evoked by stimulation in the basal ganglia (GPi and STN) have shown that short-latency, high-frequency responses, likely associated with synchronized neuronal firing, are also present in these brain nuclei (Schmidt et al., 2020; Awad et al., 2021). Here, we demonstrate the existence of long-latency, low-frequency (beta-band) neural responses in the GPi evoked by stimulation within the GPi across four patients (five hemispheres). These low-frequency, beta-band evoked responses are likely associated with synaptic currents generated within the GPi, whose synchronization results in the observed electric potentials (Izhikevich, 2006; Herreras, 2016). While the circuit dynamics underlying the generation of long-latency, beta-band ERs in the GPi are unknown, we argue that these long-latency responses are associated with the activation of multi-synaptic feedback loops in the BGTC circuit. The STN-GPe feedback loop has been implicated in the generation of spontaneous beta oscillations in parkinsonism in experimental and computational studies (Bevan, 2002; Rubin and Terman, 2004; Johnson and McIntyre, 2008; Wilson and Bevan, 2011; Alavi et al., 2022; Madadi Asl et al., 2022). A possible explanation for the generation of GPi beta oscillations evoked by GPi stimulation, which aligns with the studies cited above, is that the STN-GPe loop is antidromically activated by GPi stimulation, resulting in the onset of beta oscillations in the STN-GPe loop and the propagation of these oscillations to the GPi via the STN or GPe.

The striatum and the striatum-GPe interconnection have also been linked to the origin of spontaneous beta oscillations (McCarthy et al., 2011; Corbit et al., 2016). The stimulation-evoked beta oscillations in the GPi could emerge from antidromic activation of the striatum, resulting in the onset of beta oscillations within the striatum. These oscillations could then propagate to the GPi via the GPe and STN. While our data cannot clarify whether beta oscillations in PD originate in STN-GPe or striatal loops, future studies directed at characterizing which specific GPi neuronal elements and connections need to be activated to evoke beta oscillations in the GPi could provide insights into identifying circuitry involved in the generation of both spontaneous and stimulation-evoked beta oscillations in the basal ganglia in PD.

### 4.2. Evoked responses and PD pathophysiology

Spontaneous beta-band oscillations are associated with the manifestation of parkinsonism in animal models of PD as well as with medication- or DBS-induced changes in motor performance in PD patients (Brown et al., 2001; Brown, 2003; Brown and Williams, 2005; Kühn et al., 2006, 2009; Eusebio et al., 2009; Little et al., 2012; Escobar Sanabria et al., 2017). The ERs we identified in the studied hemispheres resonate in the beta band, where spontaneous oscillations were also observed in 4/5 hemispheres. Our data also indicate that the beta-band ERs were observed in regions of the GPi where the spontaneous beta-band oscillations were localized. This spatial correlation suggests that the same neuronal elements may be responsible for the generation of both spontaneous and stimulation-evoked beta-band oscillations in the GPi of PD patients. The spectral correlation between the spontaneous and stimulation-evoked oscillations points to the possibility that resonance—the susceptibility of a circuit to oscillate at its natural frequency—may be a fundamental mechanism underlying the generation of beta oscillations in the basal ganglia in PD. Of note, resonant, linear dynamical systems, perturbed with an impulse-like input as the electrical stimulation pulses used here, produce a response that oscillates at the system’s natural (resonant) frequencies and is damped out based on the damping associated with each natural frequency (Ogata, 1992, p. 367). Because the impulse-like inputs have components across all frequencies (i.e., the Fourier transform of the impulse is a constant across frequencies), the system’s output response elucidates the resonant frequencies of the studied system. Therefore, the impulse response can be used to characterize the resonant properties of a dynamical system operating within or close to the linear regime. Since brain circuits are dynamical systems, we argue that the stimulation-evoked oscillations reported in this study reveal the resonant frequencies of circuitry connected to the GPi. Escobar Sanabria et al. (2022) support this argument as they showed that neural responses in the GPi evoked by stimulation in the GPi and their associated resonant frequency can be characterized using linear differential equations.

### 4.3. Evoked responses as biomarkers for DBS implantation and programming

Approaches based on spontaneous or stimulation-evoked activity recorded from scalp EEG and intracranial LFPs have been proposed to identify optimal electrode contacts to deliver DBS therapy. Data from previous studies have shown that spontaneous beta-band oscillations are present in the sensorimotor region of the STN and GPi, where electrical stimulation through DBS electrodes is effective in alleviating motor signs in PD patients (Zaidel et al., 2010; Bour et al., 2015; Fernández-García et al., 2017; Petersson et al., 2020; Shah et al., 2022). Because of this spatial correlation, spontaneous beta-band activity has been proposed as a biomarker for DBS lead targeting (surgery) in the STN and programming of directional DBS leads in PD patients (Zaidel et al., 2009; Bour et al., 2015; Tinkhauser et al., 2018). Other studies have shown that stimulation of the sensorimotor region of the STN and GPi results in the generation of both short-latency, high-frequency, as well as long-latency, low-frequency ERs on scalp EEG over the MC. These studies suggest that these EEG-evoked responses can be used to define the stimulation location and thereby automate DBS programming (Walker et al., 2012; Miocinovic et al., 2018; Romeo et al., 2019). More recently, Awad et al. (2021), showed that short- and mid-latency (0.31 ± 0.1 ms and 4.5 ± 1.1 ms), high-frequency neural responses in the STN or GPi evoked by stimulation in the STN or GPi can be measured in directional DBS leads. They also showed that the amplitude of mid-latency ERs (4.5 ± 1.1 ms) in the STN or GPi is correlated with the power of spontaneous beta-band oscillations in the STN or GPi, suggesting that these ERs can also be used for DBS surgical targeting and programming. In this study, we demonstrate the existence of ERs in the GPi with much longer latency and slower frequency content than those previously reported. Additionally, we demonstrated that these ERs and spontaneous beta-band oscillations display strong spectral and spatial correlations. These spatial correlations suggest that long-latency, beta-band ERs could also be markers of DBS lead localization and programming. Because the beta-band ERs result from averaging LFP segments in the time domain, the signal-to-noise ratio of these ERs can be greater than the one of spontaneous beta oscillations. Therefore, we argue that beta-band ERs can be more robust markers for DBS lead localization than spontaneous beta-band oscillations alone. Furthermore, the long-latency, beta-band ERs shown here can be combined with stimulation-evoked responses with other latencies (e.g., mid-latency 4.5 ms ERs) and frequency content as well as spontaneous beta-band activity to build robust classification algorithms for DBS surgical targeting and programming.

### 4.4. Evoked responses to modulate spontaneous beta-band oscillations in real-time using closed-loop control

There is increasing interest in characterizing and better understanding the causal role of frequency-specific neural activity in brain disease and advancing the development of precise, patient-specific therapies. Escobar Sanabria et al. (2022), recently developed an experimental approach capable of predictably suppressing or amplifying frequency-specific (low-frequency) oscillations in the basal ganglia of nonhuman primate models of parkinsonism and patients with PD (Escobar Sanabria et al., 2020, 2022). With this technique, referred to as evoked interference closed-loop DBS (eiDBS), low-frequency neural oscillations (ERs) evoked by electrical pulses can suppress or amplify spontaneous low-frequency oscillations (synaptic related) via destructive or constructive interference when the pulses are delivered with precise amplitude and phases (Escobar Sanabria et al., 2020, 2022). eiDBS was tested postoperatively in a PD patient implanted with a directional DBS lead in the GPi (Escobar Sanabria et al., 2022). This technique could suppress or amplify GPi oscillations in the 16–22 Hz range in real-time by delivering stimulation in the GPi. Evoked responses in the GPi, mediating the modulation of eiDBS, resonated in the beta-band at the same frequency where the spontaneous beta-band oscillations were observed. The data presented in this article suggest that beta-band ERs in the GPi are present across PD patients and correlate with the location and frequency of spontaneous beta oscillations. Therefore, these data also indicate that eiDBS could be implemented across PD patients implanted with DBS leads in the GPi and support the development of clinical studies directed at understanding the degree to which controlled changes in beta-band oscillations relate to the manifestation of PD motor signs.

### 4.5. Limitations

The time when recordings were made relative to when medication was administered is inconsistent across subjects. Medications may have an impact on ER dynamics. Therefore, the ER amplitudes reported here may be under or overestimated for the studied hemispheres. In hemisphere HEMIS 1, we did not observe a peak in the beta-band of the spontaneous LFPs, which limited our ability to characterize the relationship between the ERs and spontaneous oscillations in this hemisphere. A small signal-to-noise ratio, possibly related to the location of the recording lead contacts relative to the neural sources generating the beta oscillations, could explain why the beta oscillations were observed in the ERs but not in the spontaneous LFPs. Supporting this hypothesis, the amplitude of the ERs in HEMIS 1 exhibited a small amplitude (0.1 uV) as compared to other patients whose ER amplitudes were in the 0.5–2.0 uV range. Because we averaged multiple LFP segments to obtain the ERs, we could remove noise not correlated with the stimulation pulses and thereby see the stimulation-evoked beta oscillations in the ER time series. However, we do not discard the possibility that beta band activity was not present or was negligible in HEMIS 1.

## Data availability statement

The raw data supporting the conclusions of this article will be made available by the authors, without undue reservation.

## Ethics statement

The studies involving humans were approved by the University of Minnesota Institutional Review Board (IRB#1701M04144). The studies were conducted in accordance with the local legislation and institutional requirements. The participants provided their written informed consent to participate in this study.

## Author contributions

VZ: methodology, investigation, software, validation, formal analysis, data curation, visualization, writing—original draft. JA: data collection, project logistics. LJ: data collection, data curation, writing—review and editing. JW and MH: data collection, writing—review and editing. RP: data curation, validation, writing—review and editing. CM and SC: resources, funding acquisition. DD and RM: data collection. NH: resources, writing—review and editing, funding acquisition. GM: resources, project administration, funding acquisition, writing—review and editing. MP: methodology, data collection, resources, supervision, writing—review and editing, funding acquisition. JV: resources, supervision, writing—review and editing, funding acquisition, project administration. DE: conceptualization, methodology, investigation, resources, data collection, data curation, writing—review and editing, supervision, project administration, funding acquisition. All authors contributed to the article and approved the submitted version.

## Abbreviations

BGTC: Basal ganglia thalamocortical
PD: Parkinson’s disease
GPi: Globus Pallidus internus
LFPs: Local Field Potentials
DBS: Deep Brain Stimulation
STN: Subthalamic nucleus
MC: Motor Cortex
eiDBS: Evoked interference closed-loop Deep Brain Stimulation
IPG: Implantable pulse generator
ER: Evoked response
ASD: Amplitude spectral density
PSD: Power spectral density
FDR: False Discovery Rate
