# Supplementary material for: Low-frequency deep brain stimulation reveals resonant beta-band evoked oscillations in the pallidum of Parkinson’s Disease patients
Source: Front Hum Neurosci. 2023 Sep 22;17:1178527. doi: 10.3389/fnhum.2023.1178527 (PMC10556241; doi:10.3389/fnhum.2023.1178527)
Supplement: Supplementary file 1 [file Data_Sheet_1.pdf]

Supplementary Material

Low-frequency deep brain stimulation reveals resonant beta-band evoked oscillations in the pallidum of Parkinson's Disease patients

Valentina Zapata Amaya[a], Joshua E. Aman[a], Luke A. Johnson[a], Jing Wang[a], Remi Patriat[b], Meghan E. Hill[a], Colum D. MacKinnon[a], Scott E. Cooper[a], David Darrow[c], Robert McGovern[c], Noam Harel[b], Gregory F. Molnar[a], Michael C. Park[c], Jerrold L. Vitek[a], David Escobar Sanabria[a]

[a]Department of Neurology, University of Minnesota, Minneapolis, MN, USA

[b]Department of Radiology, University of Minnesota, Minneapolis, MN, USA

[c]Department of Neurosurgery, University of Minnesota, Minneapolis, MN, USA

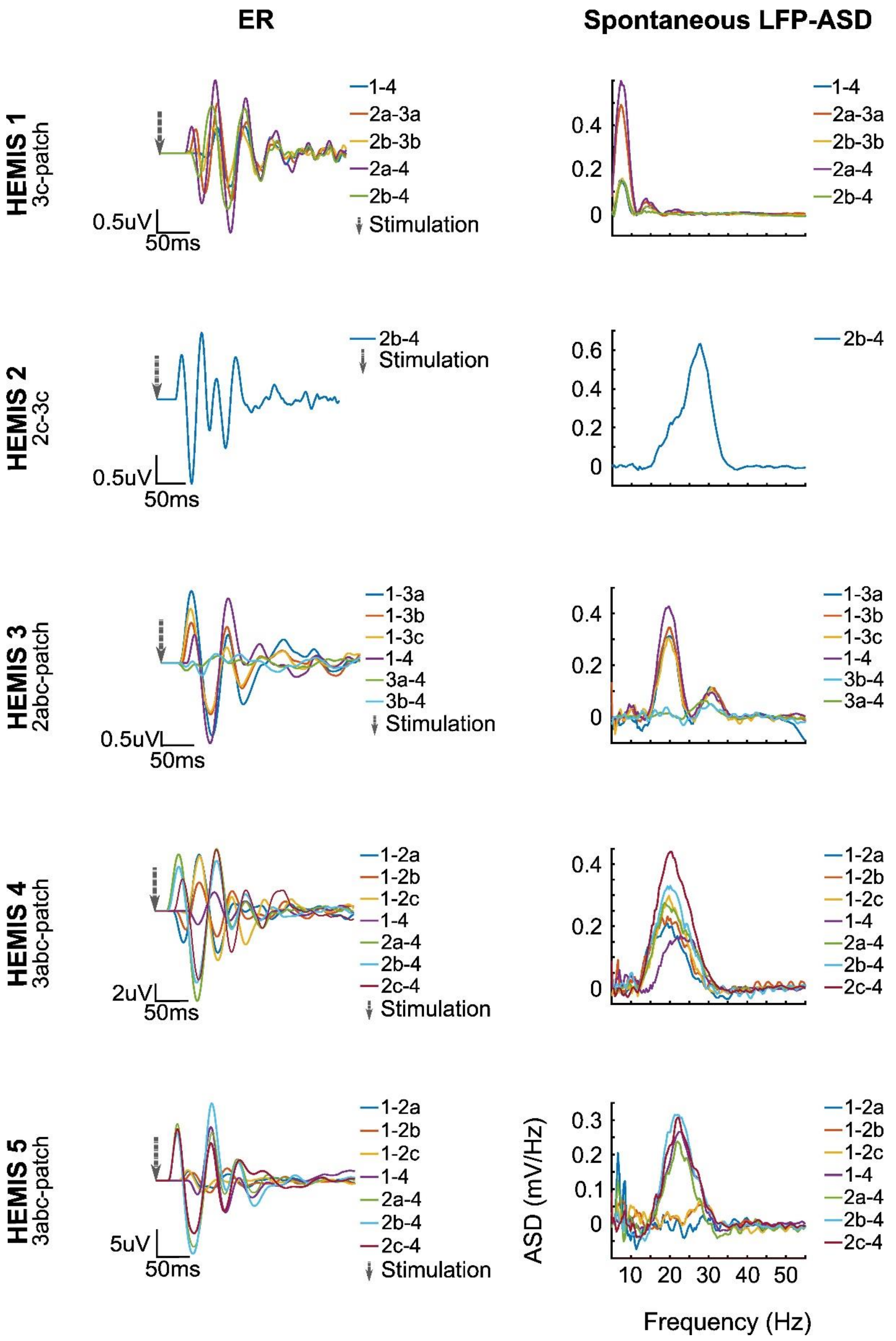

Supplementary Figure 1. ERs and spontaneous ASD curves were computed from non-stimulating bipolar configurations for all hemispheres. Stimulation montage is declared on the right side of the hemisphere label. The grey arrow in the ERs (left figures) shows the stimulation time.

Supplementary Table 1. UPDRS-III scores of the studied patients taken before the DBS implantation surgery in the off-medication condition. The hemispheres recorded with the DBS lead and UPDRS-III subscores are reported in this table.

	HEMIS 1	HEMIS 2	HEMIS 3
**Hemisphere Recorded with the DBS Lead**	**Left (right body)**	**Left (right body)**	**Right (left body)**
**UPDRS-III subscores**			
Speech	1	2	2
Facial expression	2	3	3
Rigidity: neck	3	2	2
Rigidity: RUE	2	1	1
Rigidity: LUE	1	2	2
Rigidity RLE	2	0	0
Rigidity: LLE	1	3	3
Finger Tapping: R	2	2	2
Finger Tapping: L	3	3	3
Hand movements: R	2	1	1
Hand movements: L	2	3	3
Pronation-supination movements of hands: R	2	1	1
Pronation-supination movements of hands: L	3	2	2
Toe tapping: R	2	1	1
Toe tapping: L	3	4	4
Leg agility: R	2	1	1
Leg agility: L	2	3	3
Arising from chair	1	1	1
Gait	1	2	2
Freezing of gait	0	0	0
Postural stability	1	3	3
Posture	1	1	1
Global spontaneity of movement (body bradykinesia)	2	2	2
Postural tremor of the hands: R	1	0	0
Postural tremor of the hands: L	0	0	0
Kinetic tremor of the hands: R	0	0	0
Kinetic tremor of the hands: L	0	1	1
Rest tremor amplitude: RUE	1	0	0
Rest tremor amplitude: LUE	0	0	0

Rest tremor amplitude: RLE	3	0	0
Rest tremor amplitude: LLE	3	0	0
Rest tremor amplitude: Lip/jaw	0	0	0
Constancy of rest tremor	4	0	0
**Total Score**	**53**	**44**	**44**
**Total Right**	**19**	**7**	**7**
**Total Left**	**18**	**21**	**21**
**Axial Total**	**10**	**14**	**14**

Continuation of Supplementary Table 1.

	HEMIS 4	HEMIS 5
**Hemisphere Recorded with the DBS Lead**	**Right (left body)**	**Right (left body)**
**UPDRS-III subscores**		
Speech	0	0
Facial expression	1	3
Rigidity: neck	3	3
Rigidity: RUE	1	4
Rigidity: LUE	2	4
Rigidity RLE	0	3
Rigidity: LLE	0	3
Finger Tapping: R	0	2
Finger Tapping: L	2	3
Hand movements: R	0	2
Hand movements: L	1	3
Pronation-supination movements of hands: R	0	3
Pronation-supination movements of hands: L	2	3
Toe tapping: R	0	1
Toe tapping: L	1	1
Leg agility: R	0	0
Leg agility: L	1	2
Arising from chair	0	0
Gait	0	2

Freezing of gait	0	0
Postural stability	0	0
Posture	0	1
Global spontaneity of movement (body bradykinesia)	0	2
Postural tremor of the hands: R	0	0
Postural tremor of the hands: L	2	3
Kinetic tremor of the hands: R	0	2
Kinetic tremor of the hands: L	1	2
Rest tremor amplitude: RUE	0	0
Rest tremor amplitude: LUE	2	0
Rest tremor amplitude: RLE	0	0
Rest tremor amplitude: LLE	0	0
Rest tremor amplitude: Lip/jaw	0	0
Constancy of rest tremor	2	4
**Total Score**	**21**	**56**
**Total Right**	**1**	**17**
**Total Left**	**14**	**24**
**Axial Total**	**4**	**9**
